# Advanced Glycation End Products, Diabetes, and Bone Strength

**DOI:** 10.1007/s11914-016-0332-1

**Published:** 2016-10-04

**Authors:** Masahiro Yamamoto, Toshitsugu Sugimoto

**Affiliations:** Internal Medicine 1, Shimane University Faculty of Medicine, 89-1 Enya-cho, Izumo, Shimane 693-8501 Japan

**Keywords:** Fracture, Bone quality, Material properties, Pentosidine, Crosslink, Receptor for advanced glycation end products (RAGE)

## Abstract

Diabetic patients have a higher fracture risk than expected by their bone mineral density (BMD). Poor bone quality is the most suitable and explainable cause for the elevated fracture risk in this population. Advanced glycation end products (AGEs), which are diverse compounds generated via a non-enzymatic reaction between reducing sugars and amine residues, physically affect the properties of the bone material, one of a component of bone quality, through their accumulation in the bone collagen fibers. On the other hand, these compounds biologically act as agonists for these receptors for AGEs (RAGE) and suppress bone metabolism. The concentrations of AGEs and endogenous secretory RAGE, which acts as a “decoy receptor” that inhibits the AGEs-RAGE signaling axis, are associated with fracture risk in a BMD-independent manner. AGEs are closely associated with the pathogenesis of this unique clinical manifestation through physical and biological mechanisms in patients with diabetes mellitus.

## Introduction

Diabetic patients have a higher fracture risk than expected by their bone mineral density (BMD) [1–4]. Bone strength consists of BMD and bone quality [5], suggesting that increased bone fragility in this population is, in part, caused by a deterioration of bone quality. Bone quality, conceptually, is divided into geometrical properties and material properties [6]. The former indicates the morphological characteristics of bone. Some studies indicate that aggravation of the structural properties assessed by high-resolution peripheral quantitative computed tomography (HR-pQCT) and trabecular bone score (TBS) is involved in bone fragility [7–9]. The material properties of bone, another component of bone quality, are regulated by tissue turnover, cellular activity, and oxidative stress and glycation [10]. Advanced glycation end products (AGEs) are diverse compounds that are generated via a non-enzymatic reaction between reducing sugars and the amine residues on proteins, lipids, and nucleic acids. Growing evidence of fracture risk in patients with diabetes mellitus indicates the crucial roles of AGEs in aggravating bone fragility, as in the cases of the progression of classical diabetic complications [11]. This review focuses on the material properties and summarizes the association between AGEs and bone fragility in patients with diabetes.

### Bone Mineral Density in Diabetic Patients

The measurement of BMD is an established method for assessing bone strength. An association between decreased BMD, as measured by dual-energy X-ray absorptiometry (DXA), and the fracture rate was observed in postmenopausal women with osteoporosis. In 1991, osteoporosis was defined as “a disease that is characterized by low bone mass, microarchitectural deterioration of bone tissue leading to enhanced bone fragility, and consequent increase in fracture risk” [12], and the diagnostic criteria for osteoporosis, which were primarily based on BMD, were established. In contrast, there was less information on the BMD values of diabetic patients. Diabetes mellitus is classified into two major types: type 1 diabetes mellitus (T1DM), which is caused by a loss of the ability to secrete insulin that possesses an anabolic action on bone, and type 2 diabetes mellitus (T2DM), which develops in the presence of underlying insulin resistance. In T1DM, BMD measured in the femoral neck or the lumbar vertebrae has been reported to be significantly lower than the respective value in age- and body mass index-matched non-diabetic subjects [13, 14]; these findings were consistently confirmed in other reports [15, 16]. A meta-analysis published in 2007 showed that the BMD *z* scores (the age-adjusted BMD) of hip and spine in T1DM patients were lower than the scores in non-diabetic participants [1]. In contrast, the BMD values at these sites in T2DM patients were inconsistent; early reports with a small number of patients showed that the BMD values were lower than, equivalent to, or higher than the values in the control groups [17–20]. However, successive reports from large-scale studies indicated that the BMD values in these subjects were significantly higher than the values in non-diabetic populations [21, 22]. The meta-analysis of these studies revealed that the BMD *z* scores in T2DM patients were higher than the scores in the non-diabetic population, unlike the T1DM patients [1].

### The Risk of Fracture in Diabetic Patients

The relationship between the presence of diabetes and risk of fracture has been investigated by the clinical type of diabetes. In patients with T1DM, the risk of hip fractures has been reported to be significantly higher in female patients compared with non-diabetic subjects after adjusting for confounding factors [23, 24]. Two meta-analyses confirmed the consistent relationship between the presence of diabetes and risk of fracture [1, 2]. In contrast, the findings obtained from the patients with T2DM have confused us for a long time. Some reports indicated that the risk of hip fractures is increased in T2DM. However, others showed the opposite results. Two meta-analyses concluded that the risk of hip fracture in subjects with T2DM is significantly higher than the risk in non-diabetic subjects, although their BMD was higher than the control group [1, 2]. Taken together, these findings suggest that diabetes mellitus is an underlying disease that causes secondary osteoporosis because the risk of fracture is increased in diabetic patients, irrespective of their diabetic clinical type.

### AGEs and Bone Fragility in Diabetes

- Physical effects of AGEs on bone fragility

AGE is a generic name for the various products formed from Amadori compounds by spontaneous dehydration, transposition, condensation, and oxidation reactions. Amadori compounds are generated from a non-enzymatic condensation reaction known as the Maillard reaction between amino groups and carbonyl groups, such as amino acid and reducing sugars, respectively. Thus, proteins with long half-lives, such as collagen, have more opportunities for their lateral chains to become glycated. Type I collagen is considered a major determinant factor for the material properties of bone strength because it is a prominent protein component of bone matrix. Collagen fibers are built from collagen molecules that are connected to each other through crosslinking at genetically determined site by specific enzymes, such as lysine hydroxylase and lysyl oxidase [25, 26]. Enzymatic crosslinking contributes to the improvements in tissue strength by changing the material properties of bone, such as increasing collagen stiffness [27, 28].

On the other hand, it is possible that non-enzymatic crosslinking of collagen occurs as a ubiquitous glycation process. Pentosidine (PEN) and carboxymethyl lysine (CML) are well-recognized AGEs that possess lysine or arginine residues. Because these residues in collagen fibers are used as precursor substances for the formation of these AGEs [29], PEN and CML are non-enzymatically produced in collagen fibers as crosslinked and non-crosslinked types of AGEs, respectively. In contrast to the formation of enzymatic crosslinks, an increase in non-enzymatic crosslinking by AGEs, including PEN, deteriorates bone strength. Several ex vivo studies obtained from human samples indicate that the PEN content in cancellous bone specimens from the tibia, cortical bone samples from the femoral mid-shaft and tibial mid-shaft, and trabecular bone particles from the vertebrae are inversely associated with ultimate strain, ultimate stress, and fracture toughness [30–33], with the exception of one report [34]. Indeed, the bone PEN content obtained from non-diabetic patients with hip fracture is significantly increased compared with non-diabetic patients without fracture in the clinical setting [35, 36], suggesting that an increase in the non-enzymatic crosslinked type of AGEs in collagen fibers is a well-established cause of the deterioration of bone strength. The pre-yield mechanical change in bone tissue is dominantly determined by its mineral composition; in contrast, the post-yield mechanical feature is defined by the organic matrix [37]. Indeed, several studies of non-diabetic subjects revealed that increased AGEs crosslinking was associated with reduced post-yield properties and toughness [31, 32, 38–41]. These findings suggest that AGEs potentially disturb bone strength partially through the physical influence of the excess collagen crosslinking.

The physical effects of AGEs on bone fragility are more evident in diabetic patients than in non-diabetic patients. Saito et al. showed that the bone content of PEN, a non-enzymatic crosslinked type of AGE, is significantly increased in spontaneously diabetic rats just before the onset of diabetes and that bone strength measured by the three-point bending fixture test in the diabetic group was significantly decreased compared to the control group [42]. Specimens obtained by iliac crest bone biopsy in patients with T1DM showed that the bone PEN content in subjects with fracture was significantly increased compared with subjects without fracture [43••]. PEN is a potent surrogate marker for total AGEs production because the bone PEN content reflects the total amount of AGEs in bone [44]. Accumulation of PEN in skin and bone exponentially increase with age [45, 46], and these amounts may be closely related to each other. The material strength of the bones in patients with T2DM, as confirmed by microindentation, was inferior to the non-diabetic subjects and was significantly and inversely associated with the levels of skin autofluorescence [47], indicating that the bone accumulation of AGEs aggravates the material properties of bone. Cross-sectional and prospective clinical studies showed that increased serum and urinary PEN concentrations were related to an increased risk of fractures in patients with type 1 and type 2 diabetes mellitus [48–50, 51•] (Table 1). These associations are independent of BMD. Therefore, the bone fragility of patients with diabetes, which cannot be assessed by BMD, is well explained by the poor material properties of bone caused by an increase in the levels of non-enzymatic crosslinked type AGEs, such as PEN.

**Table 1:** The association between serum or urinary pentosidine levels and fractures in patients with diabetes

	OR (95 % CI)
Vertebral fractures	
Yamamoto [48]	2.50 (1.09–5.73)*
Yamamoto [49]	1.82 (1.05–3.15)*
Schwartz [50]	5.93 (2.08–16.9)**
Clinical fractures	
Schwartz [50]	1.42 (1.10–1.83)**
Osteoporotic fractures	
Neumann [51•]	1.02 (1.00–1.03)**

Data are expressed as odds ratio of fracture after adjustment for multiple covariates per 1 unit increase in pentosidine. References [48–50], per standard deviation increase; ref. [32], per 1 pmol/mL increase.

*P < 0.05; **P < 0.01

A prospective clinical study of non-diabetic subjects showed that the circulating levels of CML are associated with hip fracture risk [52•]. Recently, the bone content of CML, a non-enzymatic non-crosslinked type of AGE, in a mouse model of type 1 diabetes was significantly increased compared with the control mice and was significantly and inversely correlated with macroscopic bone toughness, similar to PEN [53]. There is no in vitro study that revealed a significant association between an increase in the bone content of CML and a reduction of bone strength. CML appears to be the dominant AGE component. Therefore, the amount of CML may reflect the amount of PEN.

(b) Biological effects of AGEs on bone fragility

AGEs also have biological effects on bone metabolism. The receptor for AGEs (RAGE), which is presented on the surface of specific cells, recognizes AGEs as ligands [54] and is involved in the progression of diabetic complications, such as diabetic nephropathy [55]. AGEs significantly inhibit osteoblast proliferation and induce osteoblast apoptosis [56–59] and IGF-1 secretion [60]. Hyperglycemia and AGEs suppress osteoblastic differentiation and mineralization, accompanied by enhanced RAGE expression [61–63]. A reactive oxygen species (ROS) inhibitor and autophagy inducer prevent AGE-induced osteoblast apoptosis, indicating that the elevation of oxidative stress and inhibition of autophagy are involved in this event [64]. Recently, a rat model with an autograft implant containing AGEs showed that the mineral apposition rate (MAR), mineralized surface per bone surface (MS/BS), and bone formation rate (BFR) were significantly reduced, suggesting that the AGEs that accumulated in the matrix are also involved in the reduced bone formation in vivo [65]. These observations suggest that the AGE-RAGE axis plays an important role in the bone formation process. On the other hand, AGEs decreased osteoclast-induced bone resorption and the osteoclastic differentiation process [66]. AGEs increased the expression of the sclerostin protein, an antagonist of bone formation, and decreased the expression of the RANKL protein, an agonist of bone resorption, in osteocyte-like MLO-Y4-A2 cells [67]. In addition, parathyroid hormone (PTH) secretion is inhibited by AGEs and high glucose concentrations [68, 69]. These findings suggested that the pathogenesis of suppressed bone turnover, one of the characteristics of bone metabolic disorders in diabetes, is partially explained by increased AGE levels. Indeed, low bone turnover and concomitant low PTH levels are observed in patients with T2DM [70, 71] and are associated with an elevated risk of vertebral fracture [70]. This finding is independent of BMD, suggesting that bone strength is deteriorated by the poor material properties of bone due to low bone turnover caused by elevated AGE levels because tissue turnover is one of determinant factors of the material properties of bone [10].

**Fig. 1:**
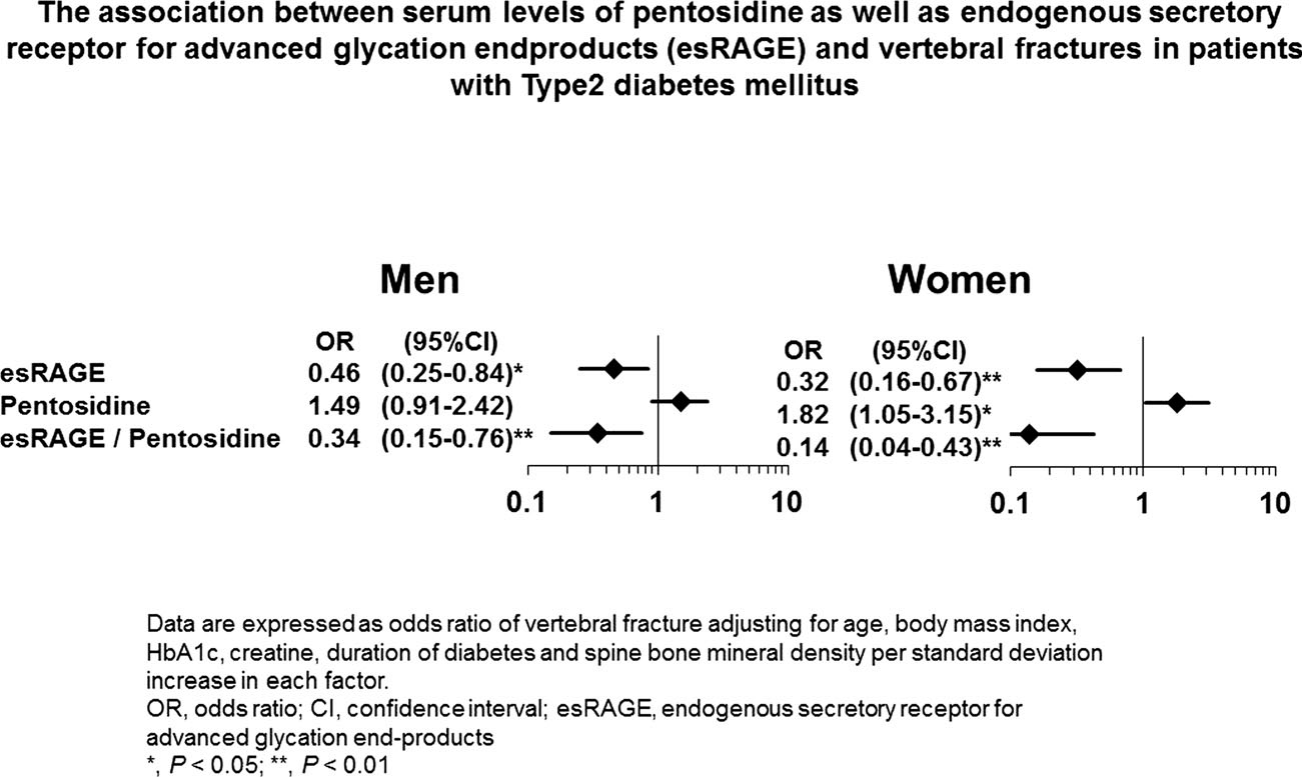
The association between the serum levels of pentosidine as well as endogenous secretory receptor for advanced glycation end products (esRAGE) and vertebral fractures in patients with type 2 diabetes mellitus. The data are expressed as odds ratios of vertebral fracture after adjusting for age, body mass index, HbA1c levels, creatine levels, duration of diabetes, and spine bone mineral density per standard deviation increase in each factor. *OR*, odds ratio; *CI*, confidence interval; *esRAGE*, endogenous secretory receptor for advanced glycation end products. *P < 0.05; **P < 0.01. Adapted from ref. [49]

Endogenous secretory RAGE (esRAGE), which is a splicing variant of RAGE that lacks the membrane-spanning portion, is known to act as “decoy receptor,” inhibiting RAGE on the cell membrane from binding to AGEs outside the cell [72]. Irrespective of sex, low esRAGE values, and relatively low esRAGE values compared to AGEs are associated with an increased risk of vertebral fractures in a BMD-independent manner in patients with T2DM who are older than 50 years (Fig. 1) [49]. This finding indicates that the AGE-RAGE signaling pathway is involved in an increase in bone fragility through the deterioration of bone quality, which may be caused by suppressed bone turnover, and indirectly suggests that AGEs biologically act as a RAGE agonist, which is engaged in bone metabolism in clinical practice. However, a recent clinical study of middle-aged patients with T1DM showed that esRAGE was not associated with the risk of fracture [51•]. These findings suggest that the preventive correlation between fracture and the esRAGE levels might be limited to older and/or T2DM patients.

### Therapeutic Effects of Anti-osteoporosis Agents on Diabetic Patient

A few clinical studies showed that some anti-osteoporosis agent drugs potentially prevent fractures in diabetic patients. When considering the pathological state of osteoporosis in diabetic patients, bone fragility in patients with diabetes may be rescued by improving the bone formation or material properties of bone. In the subanalysis of the MORE study, which examined the preventive effect of raloxifene on vertebral fracture in postmenopausal osteoporotic women, the risk of vertebral fracture after the treatment in the subgroup with diabetes was lower than that in the non-diabetic subgroup [73]. When a non-diabetic animal with elevated PEN induced by experimental diet was treated with raloxifene, bone strength recovered, presumably by decreasing the bone PEN content [74]. Therefore, raloxifene administration to diabetic patients is expected to improve the material properties of the bone matrix and prevent fractures. On the other hand, the risk of fracture in diabetic patients also increases as the BMD decreases [4]; therefore, agents that are capable of increasing BMD may be useful in preventing fractures. Teriparatide, the only current agent used to promote bone formation, decreased the bone PEN content and increased BMD in a non-diabetic animal model [75]. Teriparatide may be useful as a treatment for osteoporosis in diabetic patients because it could improve the bone matrix quality by reversing the impaired bone turnover in diabetic patients. A recent study revealed that the reduction in nonvertebral fracture incidence, increase in BMD, and decrease in back pain by treatment with teriparatide were similar in T2DM and non-diabetic patients [76]. On the other hand, bisphosphonates, which suppress bone resorption, increase BMD in T2DM patients whose bone turnover was decreased to similar degree compared to non-diabetic subjects [77], suggesting that these agents may possess particular advantages in preventing fracture in the diabetic patients with decreased BMD.

## Conclusions

The bone fragility in patients with diabetes mellitus is predominantly caused by poor bone quality because BMD is not always a useful estimate of the bone fragility of diabetic patients. AGEs are closely associated with the pathogenesis of this unique clinical manifestation through physical and biological effects on the deterioration of the material properties of bone. Further studies that clarify the etiologic mechanisms of diabetic bone fragility would provide unique diagnostic criteria and treatment strategies for this specific form of osteoporosis.
